# Perception of aesthetics and personality traits in orthognathic surgery patients: A comparison of still and moving images

**DOI:** 10.1371/journal.pone.0196856

**Published:** 2018-05-18

**Authors:** Klaus Sinko, Ulrich S. Tran, Arno Wutzl, Rudolf Seemann, Gabriele Millesi, Reinhold Jagsch

**Affiliations:** 1 University Clinic for Cranio-, Maxillofacial and Oral Surgery, Medical University, Vienna, Austria; 2 Department of Basic Psychological Research and Research Methods, Faculty of Psychology, University of Vienna, Vienna, Austria; 3 Department of Applied Psychology: Health, Development, Enhancement, Intervention, Faculty of Psychology, University of Vienna, Vienna, Austria; Medical University Graz, AUSTRIA

## Abstract

It is common in practicing orthognathic surgery to evaluate faces with retruded or protruded chins (dysgnathic faces) using photographs. Because motion may alter how the face is perceived, we investigated the perception of faces presented via photographs and videos. Two hundred naïve raters (lay persons, without maxillo facial surgery background) evaluated 12 subjects with varying chin anatomy [so-called skeletal Class I (normal chin), Class II (retruded chin), and Class III (protruded chin)]. Starting from eight traits, with Factor analysis we found a two-Factor solution, i.e. an "aesthetics associated traits cluster" and a Factor "personality traits cluster" which appeared to be uncorrelated. Internal consistency of the Factors found for photographs and videos was excellent. Generally, female raters delivered better ratings than males, but the effect sizes were small. We analyzed differences and the respective effect magnitude between photograph and video perception. For each skeletal class the aesthetics associated dimensions were rated similarly between photographs and video clips. In contrast, specific personality traits were rated differently. Differences in the class-specific personality traits seen on photographs were "smoothed" in the assessment of videos, which implies that photos enhance stereotypes commonly attributed to a retruded or protruded chin.

## Introduction

Facial appearance is the most important component of physical attractiveness [1, 2]. The first impressions a person makes are typically conveyed by his or her face. In this context rapid and subconscious assumptions based on rather meagre information are commonplace in everyday life [3]. Facial aspects like symmetry and averageness convey a number of positively perceived traits [4]. A retruded chin is a form of dysgnathia, typically perceived as baby face and as sign of social submissiveness [5], whereas a prominent chin is stereotypically associated with aggressiveness and brutality. Facial traits like attractiveness, etc. may influence career and financial success [6].

Many dysgnathic patients consider orthognathic surgery a possibility to change and improve their facial appearance. When systematically testing aesthetic and personality traits conveyed, the face presentation in video form is thought to provide more information than still images. Rubenstein [7] reported faces more attractive in video recordings, and less attractive in static video frames. Also O'Toole et al. [8] used video frames as static stimuli and found that faces and bodies in motion were perceived more favorably. Chiller-Glaus et al. [9] compared video clips and static video frames and concluded that motion has a quality of its own. However, the evidence is conflicting. Roberts et al. [10] describe factors which influence the outcome, like same or different raters, the sex of raters and/or stimuli. All these factors result in modifying the evaluation of the images. Kościński [11] reported a high correlation between still and moving images with regard to facial attractiveness. He furthermore concluded that the method of producing a face image influences the correspondence of its attractiveness with its attractiveness of the clip.

**Fig 1:**
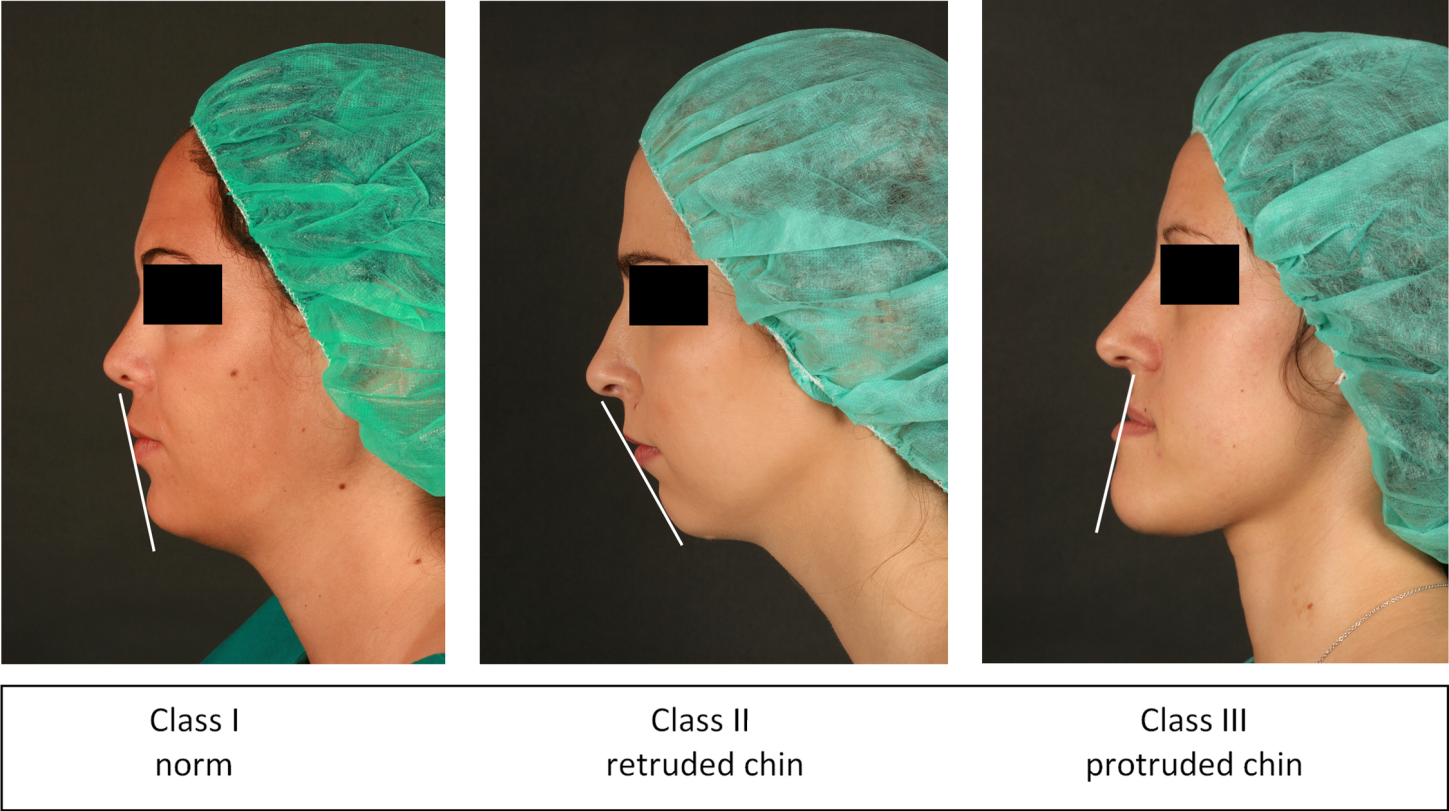
Stimuli, model persons with Class I (norm), Class II (retruded chin), and Class III (protruded chin). The persons wore no make-up, the hair was covered by a surgeons cap, to minimize distractions from the face anatomy.

Orthognathic surgeons categorize human faces based on the profile view. So-called Class I faces have the chin in the middle between Class II (retruded chin) and Class III (protruded chin; Fig 1). Skeletal Class I faces are generally considered more attractive than Class II and Class III faces [5, 12–14]. Typically the judgement of facial aesthetics includes the evaluation of patient photographs [15]. Therefore, in a previous study we analyzed the perception of images with Class II and Class III patients before and after orthognathic surgery [5]. In a second study we used short video clips instead of images [16], because moving images (video clips) may convey more aesthetics/personality related information than photographs. To contribute to the controversy whether or not faces (still or in motion) convey different impressions, we systematically compared aesthetic and personality traits conveyed by photographs and videos in patients with dysgnathic faces to explore whether or not the current practice—using photographs for the evaluation of faces—is appropriate.

## Materials and methods

### Patient recruitment

The study protocol was according to the Helsinki Declaration, and was approved by the Ethics Committee of the Medical University of Vienna (Ethics Committee approval number 1933/2014). The patients were given a detailed explanation of the study aims and its procedure (face evaluation by naïve persons), and were invited to participate in the study. The individuals in this manuscript has given written informed consent (as outlined in PLOS consent form) to publish these case details. We recruited patients with marked forms of the respective dysgnathia (Fig 1).

Because two thirds of patients who undergo orthognathic surgery are female [17], female faces were deemed more representative of the actual patients in the clinical setting. All our models were female because female faces are judged more consistently by both men and women, whereas men’s faces are assessed rather heterogeneously [18]. To minimize any bias due to signs of aging, we selected young adults between 18 and 30 years without marked signs of aging. To ensure that any possible differences in the evaluations were judged solely on visual impressions, we presented the video clips without sound by withholding vocal cues from the raters [10].

### Making of stimuli (video clips & photographs)

**Fig 2:**
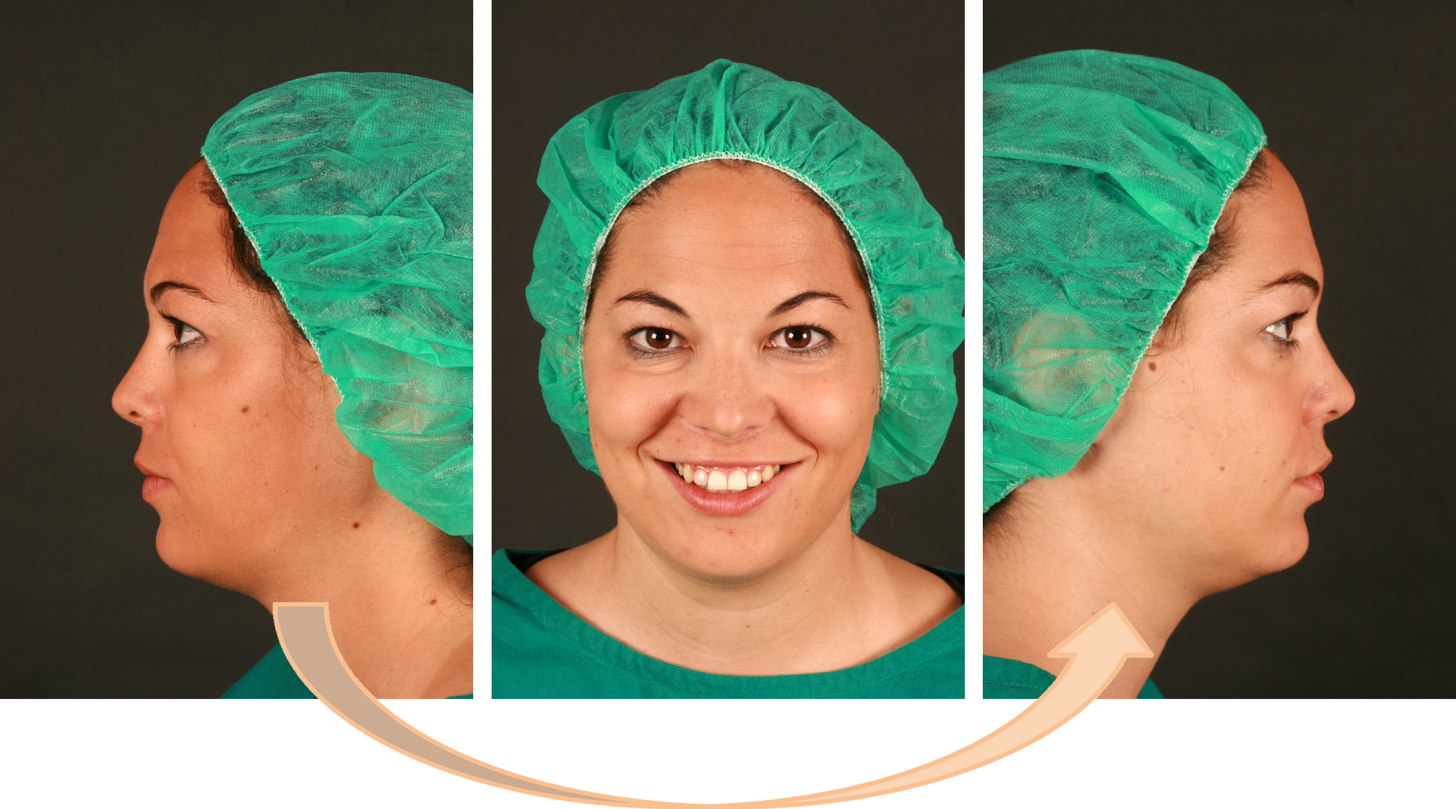
Standardized stimuli presentation of photographs. The still faces were presented to the raters from both sides and from the front simultaneously. The rotation during the video presentation was as indicated by the arrow: starting from the left profile, and slowly turned over to the right.

Four female patients from either skeletal Class II or Class III were selected as model for the stimuli production. Furthermore, we produced stimuli from four females with skeletal Class I (norm faces). From each of the 12 persons a photographic set consisting of 3 views (Fig 2) and one video clip containing the same views was produced (supplemental material S1 video). The individuals shown in the Figures and supplemental material in this manuscript have given written informed consent (as outlined in PLOS consent form) to publish their case details.

The models were uniformly dressed and positioned before a black background. The lateral views were obtained with a neutral mood expression, the frontal images showed the persons smiling. To maintain a consistent field of view, the camera distance was standardized and the photographs were taken with the same focal distance to ensure comparable picture and head sizes.

For the video recordings the study subjects were seated on a swivel chair. An assistant (K.S.), rotated the chair slowly from the left lateral view to the frontal view and then to the right lateral view. While the chair and the female study subject were being rotated for the video taping, the subject said the following out loud: “One usually says cheeeeeese on a photograph, although I’m not fond of cheese at all” (see supplemental S1 video). Photographs and video recordings were consecutively numbered, anonymized, and stored electronically in the photographic archive at the Department of Cranio-Maxillofacial and Oral Surgery.

### Raters

This study was introduced to students of the Faculty of Psychology at the University of Vienna. Each student received all the necessary information on the rating procedure, the data processing, and the publication of the study. They were then instructed to go and recruit approximately ten raters (per student) for the study. Once recruited, the raters came to the university to get information on the study protocol, and once they had all the relevant information, they were asked to give their oral consent to participate. Those who did not consent simply left, those who consented stayed and rated the images and video clips immediately. The raters were given no background information on the study subjects and dysgnathia. There were 200 raters, primarily undergraduates and graduates of various disciplines (50% females and 50% males, mean age = 24.17 years, SD = 3.56, range 19–36).

### Test design and realization

#### Evaluation setting

Roberts et al. [10] described that various design features have independent effects on the strength of static-dynamic correlations. When static and dynamic stimuli were rated by the same raters the correlations were stronger. They were weakest, if female evaluated male stimuli [10]. As per Rhodes et al. [19] we instructed the raters to assess both, the video clips and photographs spontaneously and intuitively with respect to the items (trait pairs) using a seven-point Likert scale (Table 1). The presentation material was viewed and judged in the absence of external stimuli and without interaction between the peer raters.

**Table 1:** Seven-point Likert scale for subjective rating. Each of the eight items (trait pairs) was rated along the scale.

1	2	3	4	5	6	7
Absolutely	Very much	Quite	Neither/nor	Quite	Very much	Absolutely
Item / pair No						
1		Ugly			Beautiful	
2		Unpleasant			Pleasant	
3		Unattractive			Attractive	
4		Unintelligent			Intelligent	
5		Aggressive			Good natured	
6		Inhibited			Confident	
7		Brutal			Gentle	
8		Dominant			Flexible	

## Stimuli presentation

To counterbalance the mode and sequence of presentation, one half of the raters started with the photographs followed by the video clips, the other half rated the video clips first, followed by the photographs. A five to ten minute break was maintained between the presentations of the series (photographs and videos). Class I stimuli were used as a potential anchor, each presentation series started with a Class I stimulus and continued alternating Class II and Class III. According to Rhodes et al. [19], every stimulus was followed by different dysgnathia class.

Both, the photographs and video clips were arranged in a PowerPoint^®^ presentation and were displayed to the raters on a 13 inch computer screen. After a warming up test run, a black screen appeared for five seconds, followed either by a series of video clips or photograph sets. Each video clip was announced: “The video film will start in 5 seconds”; then the standardized 7–8 second video clip started automatically.

The video presentations started consistently with the test persons´ profile from the left, followed by a rotation towards the front view—when the test person smiled briefly (“… cheeeeeese …”). The rotation continued towards the persons' profile view from the right side. At the end of the rotation, the image of the right-sided profile stayed on the screen for one more second.

All stimuli (photographs, video clips) were presented just once. The intuitive ratings were done without frozen images. For both, video clips or photographs, a single presentation and the rating documentation for the eight items took about 30 seconds.

### Rating procedure

In their research Roberts et al. [10] used the same raters for both photographs and video, every rater evaluated a face under static and under dynamic conditions. We also used the within-subjects rating design [10]. Based on their subjective intuitive perception, the raters evaluated the eight items on a given seven-point Likert scale (Table 1). To minimize systematic distortions, the raters received a folder specifying a predetermined sequence of assessment sheets. They were not permitted to scroll the pages forward or backward. The raters documented their evaluations without external influences or interaction with peers.

After the video clip faded out, the following instruction appeared: “Now please rate the person on the video clip using the eight pairs.” The raters were allowed to go through the sequences at their own pace, pressing the enter button activated the next video clip.

The sequence with photographs was introduced by the text: “We will now continue with the photo series. Please look at the photographs of each person and then give your rating after each presentation.” Then three views (left profile, front view, right profile) were shown simultaneously (Fig 2). Pressing the enter button invoked the following instruction: “Now please rate the eight pairs of characteristics.”

The evaluation tool was a list with eight items (trait pairs; Table 1), the list was specifically designed for the evaluation of patients with dysgnathia, and for the differentiation of class II and class III patients from class I [5].

### Data management and statistics

The collected data were transferred to an electronic spread sheet twice. The second entry was subtracted from the first to achieve "0" if both entries were identical. Deviations from "0" indicated a wrong entry and were corrected according to the source document. The controlled dataset was closed and used for statistical analyses with the software package IBM SPSS for Windows Version 22.

Statistical significance was defined as p ≤ 0.01. To investigate the Factor structure of the eight attributes exploratory Factor analyses were conducted using principal components analysis (PCA) with oblique rotation (oblimin). Additionally, as a measure of reliability internal consistencies (Cronbach´s Alpha) were ascertained for the items of the Factors allocated. Associations between Factor s were calculated using Pearson correlations. Gender-related comparisons of ratings of photographs and video clips were done using t-tests for independent samples. For controlling the alpha error caused by multiple testing, the method proposed by Bonferroni-Holm was used. For all comparisons that proved to be significant Cohen´s d was used as effect size. According to Cohen [20] values ≥ 0.20 are indicative for a small, ≥ 0.50 for a medium, and ≥ 0.80 for a large effect. General Linear Model (GLM) for repeated measures was applied for each Factor in order to establish whether ratings of skeletal groups differ between photographs and video clips. Thus, GLM for repeated measures used skeletal class as grouping variable and mode of presentation as the repeated variable. Additionally, gender of raters was incorporated as a covariate. In case of significant differences between skeletal classes Bonferroni post hoc tests were applied. To evaluate the effect of differences identified by means of GLM, partial eta^2^ (η^2^) was calculated, with values ≥ 0.01 indicating a small, ≥ 0.06 a medium, and ≥ 0.14 a large effect [21].

## Results

There were 12 video clips and 12 photographs, four of each Class (Class I, II and III). Evaluation of these visual media with respect to eight items (trait pairs) by 200 raters thus yielded a total of over 38.000 individual ratings. The margin of error was less than 0.05%, due to illegible or missing 20 single ratings.

Using the eigenvalue criterion, a two-Factor solution could be found for both the photo data and the video clip data, explaining a total of 75.69% (photos) and 77.91% (video clips) of the variance respectively. For both analyses, item 1 (beautiful), item 3 (attractive), item 4 (intelligent), item 2 (pleasant) and item 6 (confident) were attributed to Factor I, which we collectively refer to as "Aesthetics Traits Cluster Factor ". Item 7 (gentle), Item 8 (flexible) and Item 5 (good natured) joined to Factor II, referred to as "Personality Traits Cluster Factor" (Table 2).

**Table 2:** Results of Factor analysis. Principal components analysis (PCA) with oblique rotation; for ratings of photographs and video clips: Factor loadings, percentages of explained variance and internal contingencies for Factor I (aesthetics) and Factor II (personality).

Traits Cluster		Factor s Photo		Factor s Video	
		I	II	I	II
Aesthetics assoc. traits Cluster	Beautiful (Item 1)	**.90**	-.01	**.89**	.04
	Attractive (Item 3)	**.90**	.02	**.91**	.05
	Intelligent (Item 4)	**.80**	.08	**.86**	.03
	Pleasant (Item 2)	**.79**	.27	**.82**	.27
	Confident (Item 6)	**.74**	-.28	**.73**	-.18
Personality Cluster	Gentle (Item 7)	.08	**.91**	.13	**.90**
	Flexible (Item 8)	-.17	**.89**	-.22	**.89**
	Good natured (Item 5)	.14	**.88**	.17	**.87**
	% of variance accounted for	45.18	30.51	49.05	28.86
	Cronbach´s Alpha	.88	.88	.90	.87

Items had loadings > = .74 (photos) and > = .73 (videos) on the Aesthetics Traits Cluster Factor, and loadings < = | .28 | (photos) and loadings < = | .27 | (videos) on the respective other Factor II. Loadings on the Aesthetics Traits Cluster Factor and Personality Traits Cluster Factor on videos and photos are shown in Table 2.

Factor s appeared to be uncorrelated (r = .11) for the photo series as well as for the video clips series (r = .17). Internal consistency of the photo series data was excellent for Factor I (Cronbach´s alpha = .88) and Factor II (alpha = .88) as well as for the video clips series Factor I (alpha = .90) and Factor II (alpha = .87), respectively (Table 2).

Factor analyses separately performed for males and females replicated the two-Factor structure (73.40% accounted variance for photos and 77.19% accounted variance for videos in women, 76.89% accounted variance for photos and 78.29% accounted variance for videos in men, respectively).

Gender-related comparisons yielded significant rating differences in Factor I as well as in Factor II for both the photo series and the video clip series. Generally, women gave better ratings than men, but the effect sizes were small (Table 3). The final analysis was carried out using the General Linear Model (GLM) for repeated measures, including gender as a covariate. The GLM for repeated measures with skeletal class as grouping variable and modus of presentation as the repeated variable revealed for Factor I a highly significant result for class (F2, 590 = 135.405, p<.001, partial eta^2^ = .315), but an insignificant result for mode of presentation (F1, 590 = 2.562, p = .110) as well as an insignificant class*mode interaction (F2, 590 = 0.303, p = .739). Gender appeared to be significant (F1, 590 = 34.913, p<.001, partial eta^2^ = .056)

**Table 3:** Gender-related differences. Ratings for photographs and video clips, for Factor I (aesthetics) and Factor II (personality).

	females		males		*t*	*P*	*p'*	*d*
	*M*	*SD*	*M*	*SD*				
photo Factor I (aesthetics)	4.40	0.74	4.04	0.79	5.716	<.001*	.0025	0.47
photo Factor II (personality)	4.90	0.76	4.73	0.82	2.609	.009*	.0100	0.21
video Factor I (aesthetics)	4.30	0.79	4.06	0.82	3.733	<.001*	.0033	0.31
video Factor II (personality)	4.92	0.73	4.71	0.79	3.423	.001*	.0050	0.28

*Note*. *M* = mean, *SD* = standard deviation, *t* = *t*-score of *t*-test for independent samples, *p*´ = adjusted *p*-score after Bonferroni-Holm correction, *d* = Cohen´s *d*,

*p≤.01

**Fig 3:**
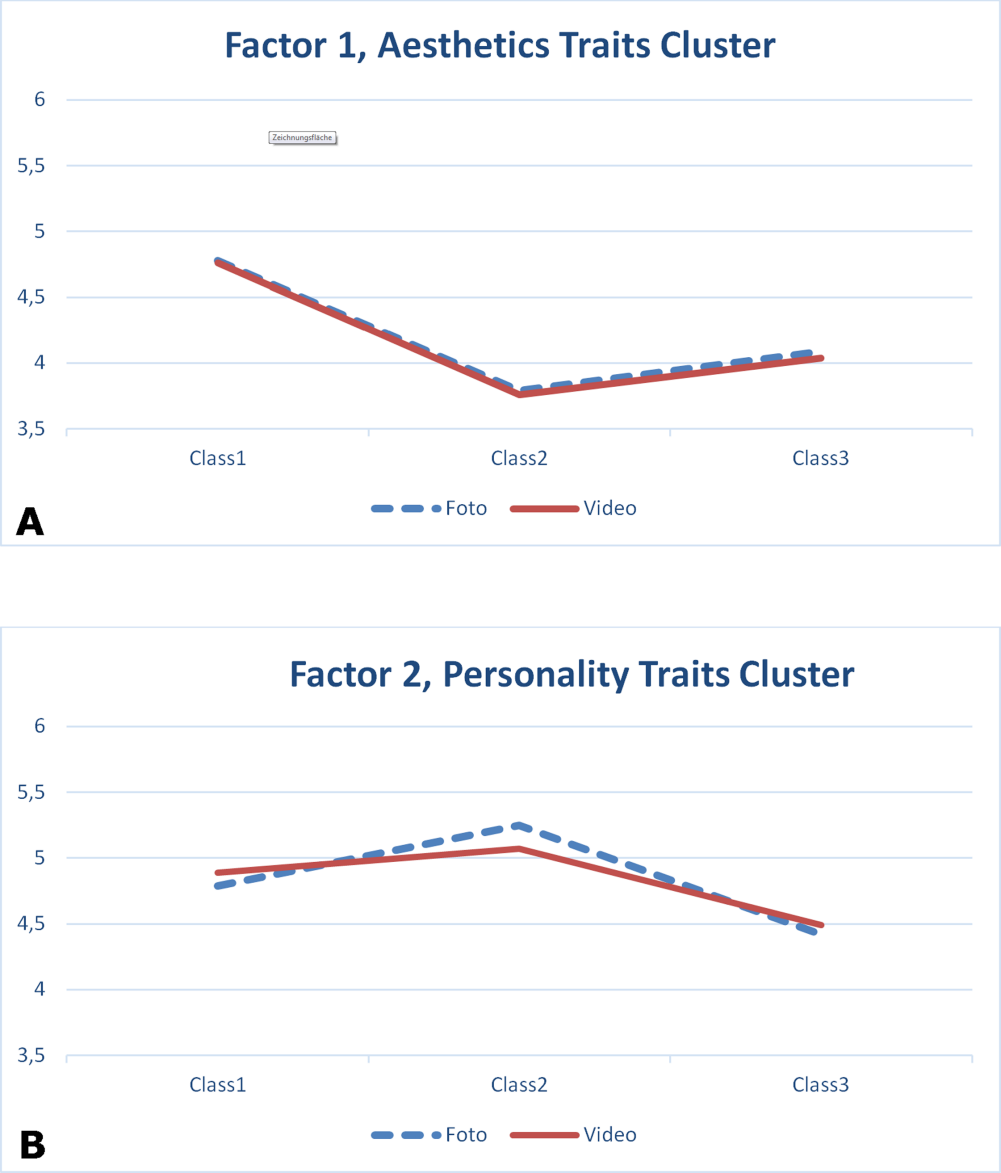
Results of GLM for repeated measures for Factor I (Aesthetics associated traits) and for Factor II (Personality). Note that for the Aesthetics Traits Cluster Factor (A) there is almost no difference between photo and video in any class. Within the Personality Traits Cluster Factor (B) the photo ratings received higher scores in Class II and lower scores in Class III, which implies that photos enhance stereotypes commonly attributed to a retruded (Class II) or protruded (Class III) chin.

Pairwise comparisons of the various skeletal classes showed that the reference group Class I appeared to have the highest ratings among the Aesthetics Traits Cluster Factor, differing significantly from Class II and Class III (p<.001). Both Class II and Class III differed significantly from each other (p<.001, Fig 3A). 

In contrast to Factor I, the interaction of class*mode (F2, 594 = 13.571, p<.001, partial eta^2^ = .044) appeared to be significant for Factor II (Fig 3B), while the mode of presentation was not significant (F1, 594 = 0.010, p = .922). Again, skeletal class showed a highly significant result (F2, 594 = 57.185, p<.001, partial eta^2^ = .161). Gender, again, appeared to be significant (F1, 594 = 12.457, p<.001, partial eta^2^ = .021). In the case of Factor II pairwise comparisons revealed that skeletal class II had the highest ratings, differing significantly both from class I and class III (p < .001), respectively, which in turn differed significantly from each other (p<.001).

## Discussion

In our study, we showed that the perception of aesthetics associated dimensions in dysgnathic faces does not change according to whether the face is presented on photographs or in video clips. Specific personality traits on the other hand, are rated differently in photographs, than they are in video clips. We observed skeletal class-dependent significant differences between photographs and videos. As previously described, photographs may enhance personality trait related stereotypes attributed to dysgnathic classes [5].

Based on experiences with similar work, we tried to minimize various sources of possible bias. Rhodes [19] concluded that inconsistences in the literature are attributable to missing standards in the presentation material (stimuli). Therefore we minimized signs of aging in the stimuli by selecting persons and raters in a very similar same age range. Furthermore our stimuli uniformly did not wear any noticeable make-up, and comparable to previous evaluations [7, 11, 19] were presented uniformly, in our case with a green surgical cap and T-shirt (Fig 2), to reduce distractions from the face.

To avoid any impact of stress, the raters were in full control of the pace of the presentations. On average the presentation of a single stimulus was for seconds, which according to Willis and Todorov [22] allows the formation of a reliable attractiveness impression after a fraction of a second. Our presentation mode also was in accordance with Ambady and Rosenthal [23], who showed that social perception of short clips closely resembles those lasting longer than half a minute.

Because Class I faces represent the social benchmark to which dysgnathic patients aspire [24, 25], it is common practice among orthognathic surgeons to approximate their treatment outcome to the anatomical features of Class I. Therefore, we started every rating session with one of the four Class I stimuli, to set a possible anchor to an orthognathic Class I face. To reduce a possible bias related to the sequence of stimulus presentation, we started the presentation session with the videos for half of the raters, and then continued with the photographs, and vice versa for the other raters.

We did not include picture material from male subjects as stimuli for three reasons. The first being that women in particular tend to rate male faces ambiguously, while more consistently rating female faces [26]. Secondly, Hönn et al. [17] showed that women’s perception of the attractiveness of male faces is very markedly influenced by her menstrual cycle. The third reason is that two thirds of all orthognathic surgery is performed on women [27], making an assessment of female faces of greater clinical relevance.

The degree to which our results can be extrapolated to the general population is limited by the raters’ age and social status, i.e. they are mostly students and their contacts aged between 18 and 36 years.

In a previous study we found that male lay raters were most critical, while male expert raters were the least critical [28]. In this study however, we found, in agreement with Nestor et al. [29], that male raters were more critical; they tended to give lower scores compared to female raters. In our study the difference between male and female raters was significant (Table 3).

The review of the literature on perception preferences between photo and video reveals conflicting results. Kościński [11] and Rhodes et al. [19] showed that there is no significant difference between the ratings of photographs and videos, while Post et al. [30] and Rubenstein [7] found that faces in videos always appear more attractive than in still images. Post et al. [30] describe the "frozen face effect" which may account for some of the discrepancies in the literature. Kościński [11] hypothesized that all studies which reported insignificant correlations between static and dynamic faces (in at least one sex), produced facial images from a clip frame rather than taking a separate photograph [7, 31, 32]. This suggests that the method of producing a face image influences the correspondence of its attractiveness with the attractiveness of the clip [11]. Our findings corroborate Lander [31] and Penton-Voak and Chang [32] who found high image-clip attractiveness concordance for female faces, and also substantiate Diener et al. [33] who found a strong correlation between attractiveness of faces seen on photos and on films.

When investigating the Factor structure of the eight items using principal components analysis, the eight traits were attributable to two Factor s. We identified two trait cluster Factor s: (1) aesthetics traits and, (2) personality traits. It is known that the trait "intelligence" closely follows aesthetic trait patterns [34]. It was a surprise to find the trait pair inhibited/confident in the aesthetics associated Traits Cluster Factor as well (Table 2).

The fact that in the aesthetics-associated Traits Cluster Factor Class II scored lower than Class III (Fig 3) may reflect changes in beauty ideals that have occurred over recent decades. While a retruded chin was considered a female beauty ideal in the first half of the last century, a straight profile with a rather dominant chin has been deemed attractive since the 1980s.

In other words, persons with a retruded chin (Class II) have the status of being rather good natured, flexible and gentle, while persons with a prominent (protruded) chin (Class III) tend to be regarded as having the opposite character traits [5]. In this respect our data are consistent with the literature. Considering the traits, we saw no difference in the perception of aesthetic traits when the stimuli were photographs or videos (Fig 3A). For the aesthetics-associated traits cluster photographs do not cause any differences in the ratings, compared to videos.

Considering the Personality Traits Cluster, we saw a difference between photographs and videos (Fig 3B). Photographs enhanced stereotypes and increased the distances between the classes; the ratings of video clips revealed lower distances between the classes and decreased the perception along stereotypes.

Since the planning of an orthognathic surgery intervention routinely uses photographs, our findings confirm the validity of this standard.

## Conclusion

Aesthetic traits seem to be perceived with no or little difference between photo and video. For personality traits photographs accentuate common stereotypes associated with Class II and Class III dysgnathia. There is no need to change common practice; the use of photographs for evaluation of dysgnathic faces is appropriate.

## Supporting information

S1 Video Stimulus as presented to the raters. (AVI)
